# Minimally invasive debridement and drainage using intraoperative CT-Guide in multilevel spondylodiscitis: a long‐term follow‐up study

**DOI:** 10.1186/s12891-021-03988-1

**Published:** 2021-01-29

**Authors:** Jianbiao Xu, Leiming Zhang, Rongqiang Bu, Yankang Liu, Kai-Uwe Lewandrowski, Xifeng Zhang

**Affiliations:** 1 Department of Orthopaedics,First, Affiliated Hospital of Tsinghua University(Beijing Huaxin Hospital), Beijing, China; 2 School of Clinical Medicine, Tsinghua University, Beijing, China; 3 Department of Neurosurgery, The Sixth Medical Center of PLA Hospital, Beijing, China; 4 Beijing Yuhe Orthopaedics Hospital, Beijing, China; 5 Shanxi Medical University, Taiyuan, China; 6 Center For Advanced Spine Care of Southern Arizona, Surgical Institute of Tucson, Tucson, USA; 7 Department of Orthopaedics, Beijing Tsinghua Changgung Hospital, School of Clinical Medicine, Tsinghua University, Beijing, China

**Keywords:** Spondylodiscitis, Minimally invasive surgery, Drainage

## Abstract

**Background:**

Spondylodiscitis is an unusual infectious disease, which usually originates as a pathogenic infection of intervertebral discs and then spreads to neighboring vertebral bodies. The objective of this study is to evaluate percutaneous debridement and drainage using intraoperative CT-Guide in multilevel spondylodiscitis.

**Methods:**

From January 2002 to May 2017, 23 patients with multilevel spondylodiscitis were treated with minimally invasive debridement and drainage procedures in our department. The clinical manifestations, evolution, and minimally invasive debridement and drainage treatment of this refractory vertebral infection were investigated.

**Results:**

Of the enrolled patients, the operation time ranged from 30 minutes to 124 minutes every level with an average of 48 minutes. Intraoperative hemorrhage was minimal. The postoperative follow-up period ranged from 12 months to 6.5 years with an average of 3.7 years. There was no reactivation of infection in the treated vertebral segment during follow-up, but two patients with fungal spinal infection continued to progress by affecting adjacent segments prior to final resolution. According to the classification system of Macnab, one patient had a good outcome at the final follow-up, and the rest were excellent.

**Conclusions:**

Minimally invasive percutaneous debridement and irrigation using intraoperative CT-Guide is an effective minimally invasive method for the treatment of multilevel spondylodiscitis.

## Background

Spondylodiscitis is an unusual infectious disease, which usually originates as a pathogenic infection of intervertebral discs and then spreads to neighboring vertebral bodies [1]. Mortality is around 2–3 % [2]. Its incidence varies between 0.2 % and 3.6 % after spine surgery [1, 3, 4]. There is no uniform standard for the treatment of spondylodiscitis. Conservative therapy including bracing and appropriate antibiotics is always adequate for the patients involving early detection of mild infection [5]. But delayed diagnosis and treatment of spondylitis are common because of their early variable clinical manifestations and indolent courses, which may lead to the failure of conservative treatment [5]. Surgical treatment is reserved for patients with failed conservative therapy, including intractable spinal pain, large epidural abscesses, and extensive vertebral body destruction [6]. The major purpose of surgical intervention in spondylodiscitis is to remove the infected tissues, relieve spinal pain, rebuild the spinal stability, and improve limb dysfunction. Open surgery has been advocated in the past [7–11]. However, whether the anterior or posterior approach, open surgery faced serious complications like nerve or vessel injuries due to extensive anatomical dissection and destruction of the stable spinal structure [12, 13]. Recent research favors a minimally invasive surgery(MIS) [7, 14, 15].

Existing studies have focused on single-level or early-stage infectious spondylodiscitis, and good clinical results have been reported after percutaneous debridement and drainage [14, 16, 17]. However, there are few reports of the management of advanced multilevel infections. These are difficult to treat using open or endoscopic surgery in current clinical practice, because of mechanical instability of the affected multilevel segments caused by widespread destruction due to the disease process [14, 18–21]. To my knowledge, up to date, this is the first report to treat multilevel spondylodiscitis with MIS. MIS may provide minimized damage stable structure of the posterior spine and paraspinal soft tissues. However, it is difficult to identify anatomic landmarks in MIS that may lead to severe complications. Therefore, to increase the accuracy of debridement and drainage, CT-Guide was performed during the operation.

The purpose of this study was to evaluate the clinical effect of percutaneous debridement and drainage using intraoperative CT-Guide in the treatment of multilevel spondylodiscitis. Considering the particularity of tuberculous spondylodiscitis, it was not included in this study.

## Methods

### Patients

From January 2002 to May 2017, 23 patients with multilevel spondylodiscitis were treated with percutaneous debridement and drainage procedures in our department. There were 12 female and 11 male patients with an average age of 56.5 years (range from 40 to 65 years). All patients were treated conservatively (antibiotics and bed rest) or with open surgery in other hospitals before transfer to our department. There were 7 cases of infection after open or minimally surgery and 16 cases of unknown etiology.

Clinical diagnosis of spondylodiscitis was mainly based on routine blood tests including C-reactive protein (CRP), and erythrocyte sedimentation rate (ESR); imaging examinations comprising X-ray, CT scan, and magnetic resonance imaging (MRI).

### Operative procedure

The patients were positioned in the prone position after induction of local anesthesia on a radiolucent surgical bed. Under CT-Guide (Brainlab® System), the target disc was located and the entry site was marked on the skin at a point 3–10 cm from the midline. All cases were treated by a transforaminal approach. The puncture direction was about 45 degrees abduction. The needle was punctured through the safety triangle to the infected vertebral space. On each side, a spinal needle was inserted directly into the infected disc and through the spinal needle, a guidewire was introduced. After a small incision (about 1 cm) was made, a dilator and a cannulated sleeve were guided through the wire into the targeted site. The infected tissue of the targeted disc was extracted with discectomy forceps through the cannulated sleeve if necessary. The same procedures were performed on the contralateral side. In the case of a paravertebral abscess, paravertebral tube drainage should be performed at the same time. This allowed for both sufficient biopsy material and extensive debridement of the necrosis and inflammatory tissue from a different direction. After biopsy and debridement, at least 1000 ml of the physiological saline was used for irrigation at each level. Finally, double cavity flushing drainage catheters were placed into the debrided segment and attached to the negative pressure suction for postoperative irrigation. Postoperatively, 1500 ml of broad-spectrum antibiotic saline was used irrigated locally every day via continuous irrigation and flushing. After the results of microbial culture became available, broad-spectrum antibiotics were replaced by those with more narrow-sensitivity. The antibiotic treatment in the perioperative period follows the relevant literature [5].

The criteria for the cessation of irrigation was: 1. complete disappearance of clinical symptoms; 2. clear fluid following flushing; 3. CRP declined to the normal range or the level before spondylodiscitis. If two of the above three indicators are met, the flushing will be stopped. After cessation of irrigation, the cannulae were removed after about 48 hours if CRP remained low.

### Outcome measures

All patients were followed up in the clinic at 1 month and then every 3 months to determine whether the infection remained under control after discharge [22]. All patients were followed up with X-ray and MRI at each visit [23]. The following factors were assessed: physical examination, laboratory tests, back pain score (visual analog scale, VAS), Oswestry disability index(ODI), and Macnab criteria(as proposed by Macnab I) [22]. After the operation, the patient was asked to identify which one of the four levels corresponded to their condition: excellent, good, fair, poor. No pain and no restriction of activity are excellent; occasional back or leg pain is good; intermittent pain affecting work and life is fair; no improvement or further operative intervention required is poor.

### Statistical methods

SPSS 23.0 was used for statistical analysis. Overall summary statistics were calculated in terms of means ± SD for continuous variables. In this study, t-test was used for measurement data. All statistical tests were bilateral, with P < 0.05 as the significance standard.

## Results

### Demographic data

The 23 enrolled patients included 9 patients with Gram-positive (+), 6 patients with Gram-negative (-), 7 patients with Fungi, and 1 with mixed infection (Tables 1 and 2). Of the 23 patients, the maximum number of infected levels was 6 and the average number of infected levels was 3.2.

**Table 1:** Cultured pathogens in 23 patients received minimally invasive debridement and drainage treatment

Cultured pathogens	Number
Gram-positive (+)	9
S. aureus	4
S. epidermidis	3
S. viridans	1
MRSA	1
Gram-negative (-)	6
E. coli	3
E. faecalis	2
Brucella	1
Fungi	7
Aspergillus fumigatus	5
Aspergillus flavus	2
Mixed infection	1
Aspergillus fumigatus and S. aureus Total Number	1 23

**Table 2:** Demographic data of seven patients with postoperative spinal infection

NO	Operations before MIS	Internal fixation (Y/N)	Fixation removed (Y/N)	Cultured bacteria
Case 1	open lumbar surgery + debridement and internal fixation	Y	N	Aspergillus fumigatus
Case 2	Open internal fixation of lumbar spine	Y	N	Aspergillus flavus
Case 3	Open Operation of malignant schwannoma	N	-	E. coli
Case 4	Open internal fixation of lumbar spine	N	-	S.epidermidis
Case 5	Open internal fixation of lumbar spine	Y	N	S. aureus
Case 6	Unidentified Lumbar infection + open debridement and internal fixation	Y	N	Aspergillus fumigatus
Case 7	Open internal fixation of lumbar spine	Y	N	S. aureus

At admission, all but one patient presented with a fever of more than 38.5 °C. All patients presented with ESR of more than 20 mm/hr (range, 50 to 115 mm/hr). The elevated CRP ranged from 14.9 mg/L to 30.6 mg/L with an average of 23 mg/L (Table 3). The white blood cell count was elevated above normal in only one case.

**Table 3:** The results of the outcome measures for Clinical and laboratory indicators

Laboratory tests	Pre-Treatment (at admission)	Post-Operation (1w)	Post- Extubation (1 m)
ESR (mm/hr)	84.5 (50–115)	23.4 (15–38)	13.2 (6–19)
CRP (mg/L)	26.0 (14–30)	16.5 (12–21)	3.7 (2–10)
Functional results			
Visual analog scale	8.6 (6–10)	4.5 (3–5)	2.3 (1–3)
Oswestry disability index	69.3(56–89)	34.2 (25–44)	26.3 (13–42)

In this study, 7 patients with spondylitis were infected after the operation, and 5 of them underwent internal fixation implantation. All patients received minimally invasive treatment without the removal of internal fixation (Table 2). Before coming to our hospital, only 2 patients had a culture report (fungal infection), and both of them underwent open debridement surgery in other hospitals, and then transferred to our hospital.

### Radiologic findings

**Fig. 1:**
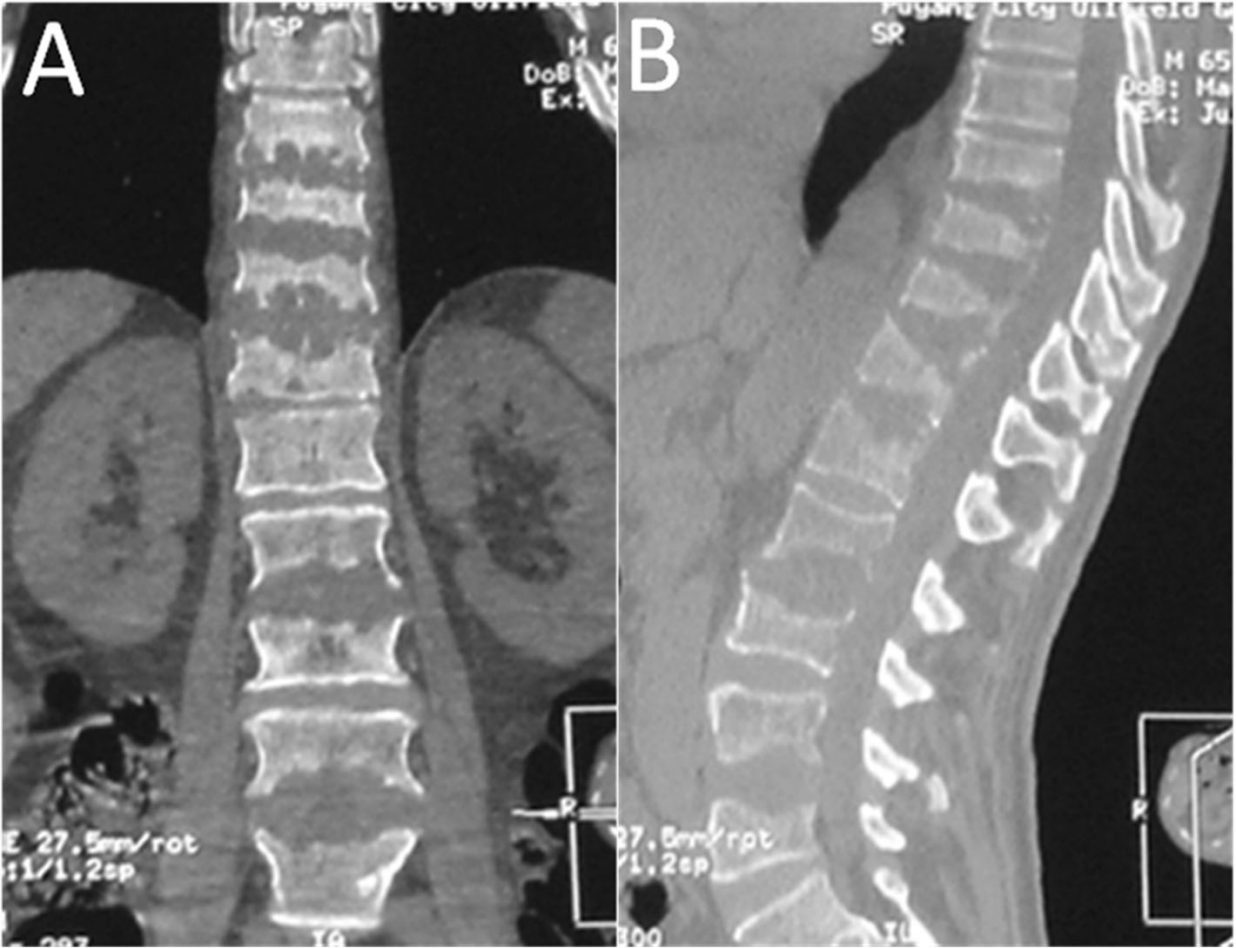
A 56-year-old man was diagnosed as having T9-12/L2-3/L4-5 fungal spondylodiscitis. The CT scan showed the endplates were grossly destroyed

**Fig. 2:**
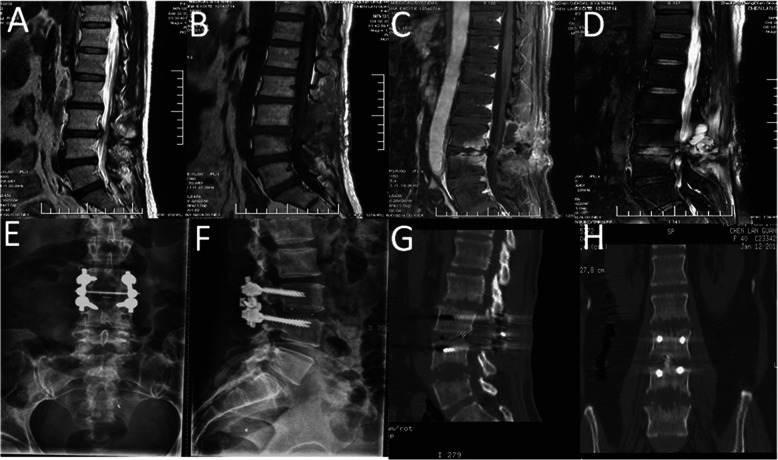
Sequential radiologic findings in a previously healthy 40-year-old man (case 1). Prior to primary lumbar discectomy, sagittal T2-weighted MRI showed a herniated disk at L3/4 (**a**). The original single-level infection was detected by T1-weighted MRI, approximately 2 weeks after primary discectomy (**b**). One month later, T1- and T2-weighted MRI showed the infection had progressed to L3/4 (**c**) and (**d**). Lesion debridement + bone graft fusion + pedicle screw fixation were performed in another hospital(**e**) and (**f**). CT showed that the infection had progressed to the adjacent L2/3 and L4/5 levels, with endplate destruction 2 months after fixation surgery(**g**) and (**h**)

In all patients, X-ray examination showed no signs of spinal instability; CT showed destruction of the vertebral endplate and varying degree of vertebral space collapse (Fig. 1). MRI revealed no compression of the spinal cord. There was no recurrence in the treated vertebral segment during follow-up, but two patients with fungal spinal infection (Case1 Fig. 2/3) (Case2 Fig. 4/5) progressed to involve adjacent segments. During postoperative follow-up, no deformities such as scoliosis or kyphosis were observed by plain radiography, and MRI showed varying degrees of spontaneous fusion [24].

**Fig. 3:**
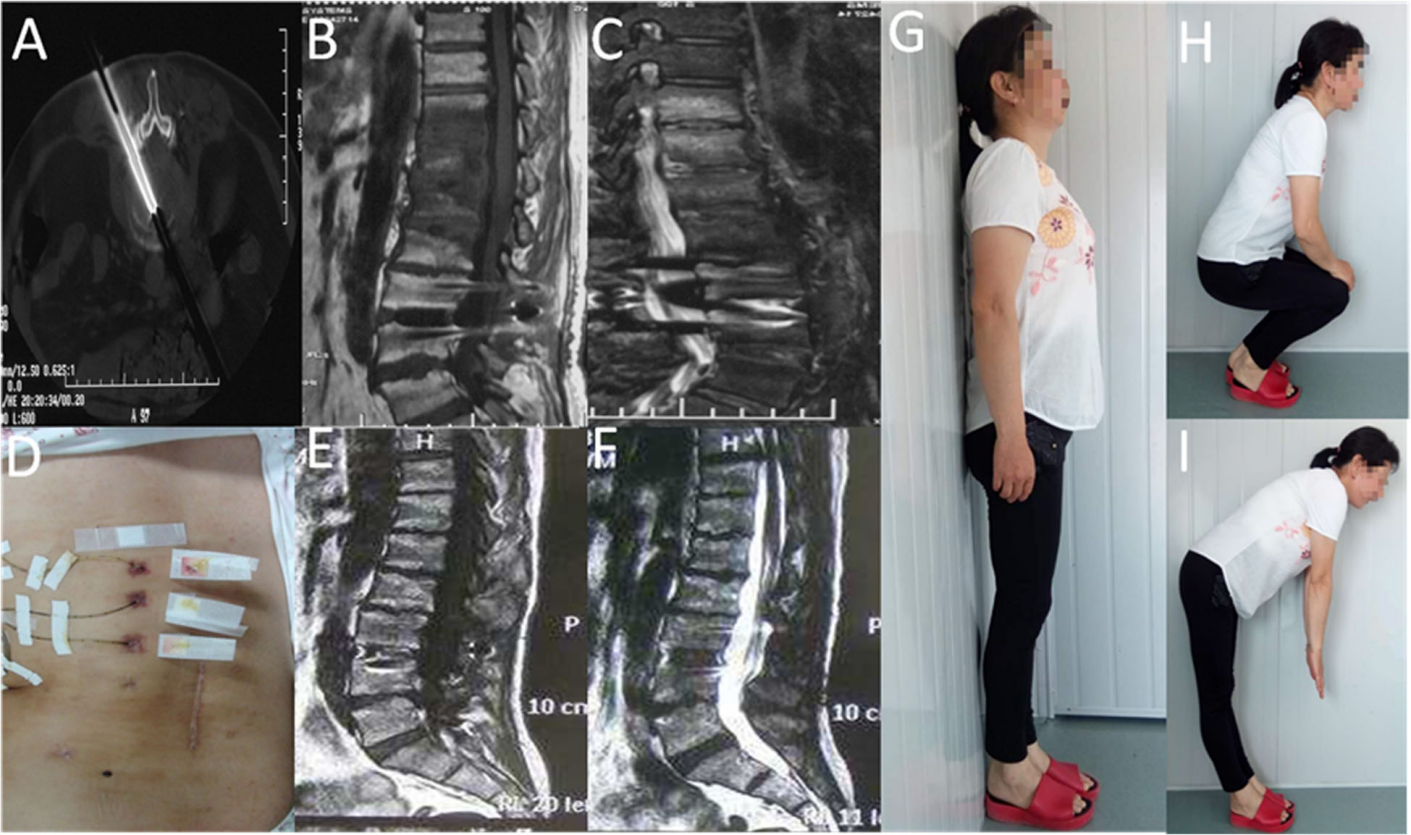
The same 40-year-old female as shown in Fig. 2. Samples confirmed a fungal spondylodiscitis (case 1) using intraoperative CT-Guide(**a**). At five months after the minimally invasive debridement and irrigation, MRI showed that the L2-5 infections had resolved but the infection had progressed to the L1-2、T12-L1 and T11-12 levels (**b** and **c**). Postoperative minimally invasive debridement and irrigation picture(**d**). At the 48-month follow-up, MRI (**e**) and (**f**) showed all the infections had resolved and no further progression was observed; T1-weighted image showed increased signal with the extensive additional enhancement of the fat signals of vertebral bodies. The endplates were grossly deformed. T1 and T2-weighted show a narrow line at the site of the discs

**Fig. 4:**
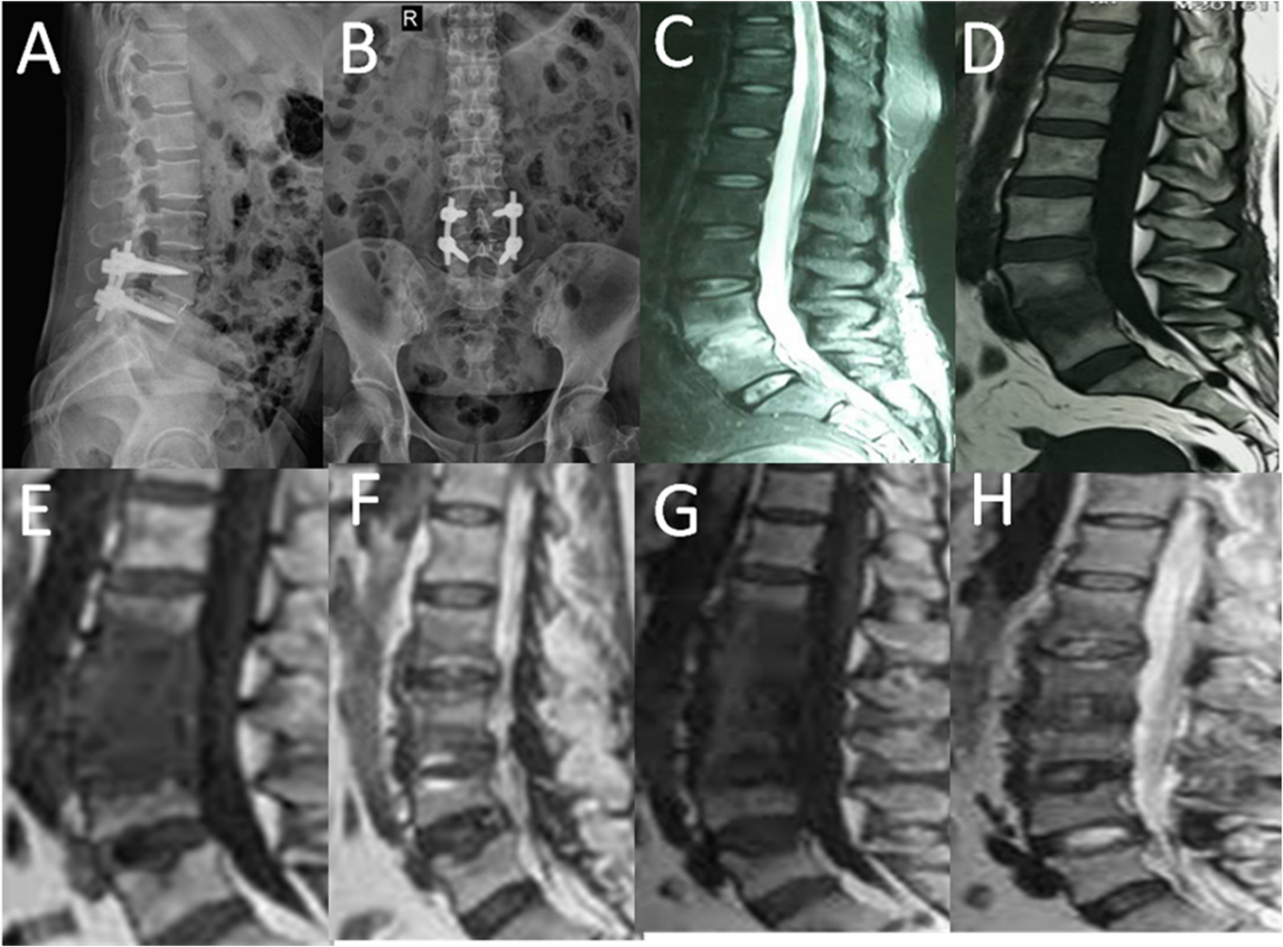
Sequential radiologic findings in a previously healthy 49-year-old woman before admission to our institution (case 2). X-ray showed L4/5 internal fixation and fusion surgery (**a** and **b**). The original single-level infection(L4/5) was detected by MRI, approximately 40 days after the primary operation (**c** and **d**). Three months later, T1- and T2-weighted MRI showed the infection had progressed to L2/3 and L3/4 (**e**) and (**f**). Two months later, T1- and T2-weighted MRI showed the infection had progressed to L1/2 (**g**) and (**h**)

**Fig. 5:**
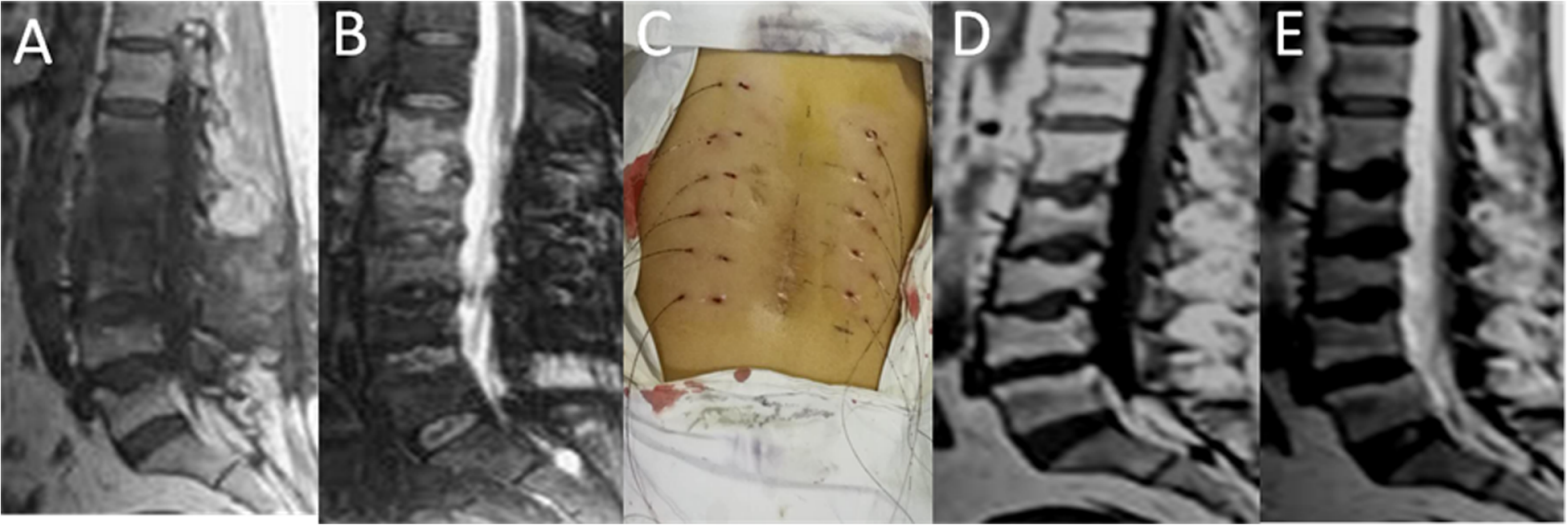
The same 49-year-old female as shown in Fig. 4 (case 2). Lesion debridement + fixation removal was performed. MRI in our institution showed that the infection had progressed to the L1/L2 level, with endplate destruction six weeks after fixation removal (**a**) and (**b**). Clinical photograph of postoperative percutaneous debridement and irrigation (**c**). At 12-month follow-up, MRI T1(**d**) and T2(**e**) showed all infection had resolved

### Clinical outcomes

Before surgery, all patients had significant back pain. There were 2 patients with radiating lower limb pain but no patients had muscle weakness, bowel or bladder dysfunction preoperatively. The operation time ranged from 30 minutes to 124 minutes for every spinal level with an average of 48 minutes. Intraoperative hemorrhage was minimal. In this patient population, the average delay of the diagnosis[25] was thirty-seven days. There were significant differences in VAS and ODI between the pre-treatment and post-operation (P > 0.05). And there were significant differences in VAS and ODI between the pre-treatment and post-extubation (*P* > 0.05) (Table 3).

The average drainage time was 14 days (5–26 days). No serious complications were found in the perioperative period. In one case (Case 6), a drainage tube became detached, and another puncture and catheterization were performed.

### Follow‐up

Postoperative follow-up periods ranged from 1 year to 6.5 years, (mean 3.7 years). One patient (Case 2) was lost to follow-up at postoperative 12 months. One patient (Case 3) died from an abdominal neoplasm at postoperative 12 months. According to the classification system of Macnab [22], one patient (Case 2) had a good outcome at final follow-up, and the remainder were excellent.

## Discussion

Spondylodiscitis is a term encompassing vertebral osteomyelitis, spondylitis, and discitis, the incidence of which is increasing due to an increase in the susceptible population and improved diagnostics [26, 27]. The etiology of spondylodiscitis usually is monobacterial infection and in Europe,more than 50 % of cases are caused by Staphylococcus aureus [5, 28]. Fungal discitis is rare and to date, only case reports exist [26]. Fungal discitis is usually due to molds [29–32] and Candida species [33–38], which are consistent with our results.

Clinical presentation of spondylodiscitis especially early stage may be nonspecific. Refractory and unremitting back pain often requiring narcotic pain control is the most common clinical presentation, with fever and neurologic deficiency less frequently encountered [1, 39]. The clinical symptoms appear at an average of six weeks after the primary procedure [1]. Elevated ESR and CRP are extremely sensitive, but WBC count might be within the normal range [40]. In our series, CRP and ESR values were increased on admission in all patients, whereas white blood count was increased in only one patient. MRI is the gold standard in imaging studies to detect spondylodiscitis [5, 41]. MRI exhibits high specificity and sensitivity,which are extremely high at 96 % and 92 % respectively [23, 42, 43]. Therefore, we routinely performed an MRI during the follow-up.

Treatment of spondylodiscitis, especially fungal infection, is often delayed because fungal organisms are slow-growing and difficult to identify by culture [25]. CT-guided needle biopsy has been recommended for the isolation of causative pathogens [44–46]. However, the aspirate is often inadequate. Percutaneous endoscopic aspirate has been reported to have a high accuracy rate in the detection of causative organisms [47]. In this study, the causative organisms were extracted through a cannula less than 1 cm diameter using discectomy forceps. Our study uses CT-Guide so the discectomy forceps were able to access the center of the lesion. Although the radiation dose of CT is higher than that of C-arm, CT-Guide is accurate and convenient for precise catheterization. We also consider that careful CT-Guide also provides potential benefit for theatre-users by reducing radiation exposure compared with fluoroscopically assisted spinal surgery [48, 49].

Multilevel spondylodiscitis after surgery is an intractable and troublesome complication, which may not be resolved by simple surgical debridement. Major open surgery has important drawbacks in patients with multilevel infection because of concern that the extensive destruction and mechanical instability of the affected segments following this type of surgery may be associated with significant rates of perioperative complications and mortality [1, 50]. Minimally invasive endoscopic debridement with dilute betadine solution irrigation is an effective alternative to extensive open surgery for the treatment of single-level infectious spondylodiscitis,but the effectiveness of this procedure for extensive destruction of vertebral bodies and multilevel refractory infections may be limited because the thorough debridement of synchronous multiple lesions by endoscopic means is difficult and can exhaust both patient and surgeon [14, 15, 51, 52]. Minimally invasive drainage and continuous irrigation with local administration of antibiotic agents, including minimally invasive suction aspiration, have been found to be effective in patients with spondylodiscitis [53, 54].

There are several advantages of our method: (1) Continuous drainage can dilute the density of pathogens, which reduces pathogenic capacity (including the invasiveness of pathogens, external toxins, and endotoxins); (2) Minimally invasive implant of the drainage tube will not cause major surgical trauma and is conducive to the rehabilitation of patients; (3) Continuous perfusion does not destroy the body’s protective immune response; (4) Formation of hematoma as a culture medium is inhibited [12].

In our series, two patients with fungal infection showed progressive disease spreading to adjacent segments. A previous study has reported this unique pathological feature of spondylodiscitis, but the exact reason remains unclear[39]. It may depend on the premorbid general condition of the patient (malnutrition, immune suppression, malignancy), the type of fungal species, and delay in treatment[55]. Progressive disease may occur either above or below the lesion. Adjacent segment preventive catheterization was performed in one patient and achieved its purpose, but its reliable effect needs further study.

This study has several limitations. First, we were able to collect only 23 cases; firmer conclusions await large sample studies. Second, because our study was a retrospective non-control study of various disc infections, it is difficult to evaluate the efficacy of surgery independent of antimicrobial therapy. The feasibility and benefits of minimally invasive debridement and irrigation for the treatment of multilevel spondylitis need to be further evaluated in larger series as part of a prospectively controlled study. Third, in view of the particularity of tuberculosis treatment, the patients with tuberculosis spondylitis were not included in this study. In addition, this technology can’t be used for decompression and deformity correction. For patients with neurological compromise or spinal deformity, open surgery would be the requirement. However, in our cohort, enrolled patients had similar clinical features of multilevel spondylodiscitis and all operations were performed by the same experienced surgeon (XFZ).

## Conclusions

On the basis of these study findings, we believe that minimally invasive debridement and irrigation using intraoperative CT-Guide is an effective minimally invasive method for the treatment of multilevel advanced spondylodiscitis.

## Data Availability

The datasets used and/or analysed during the current study are available from the corresponding author on reasonable request.

## Abbreviations

**CRP**: C-reactive protein.
**ESR**: Erythrocyte sedimentation rate
**MRI**: Magnetic resonance imaging
**MIS**: Minimally invasive surgery
**VAS**: Visual analog scale
**ODI**: Oswestry disability index
